# Oxidative Stress and Nutritional Antioxidants in Renal Diseases: A Narrative Review

**DOI:** 10.3390/antiox14070757

**Published:** 2025-06-20

**Authors:** Dorin Dragoș, Iulia I. Enache, Maria M. Manea

**Affiliations:** 1 Faculty of Medicine, Department 1–Medical Semiology, Nephrology–Bucharest Emergency University Hospital, ‘Carol Davila’ University of Medicine and Pharmacy, 050474 Bucharest, Romania; dorin.dragos@umfcd.ro; 2 Doctoral School, Faculty of Medicine, ‘Carol Davila’ University of Medicine and Pharmacy, 050474 Bucharest, Romania; 3 Faculty of Medicine, Department 6–Neurology–National Institute of Neurology and Neurovascular Diseases, ‘Carol Davila’ University of Medicine and Pharmacy, 050474 Bucharest, Romania; maria.manea@umfcd.ro

**Keywords:** acute kidney injury, chronic kidney disease, glomerulopathy, aging kidney, nephrolithiasis, natural nutrients, oxidative stress

## Abstract

Oxidative stress is a key component in the pathogenesis of a broad number of renal disorders, including acute kidney injury, chronic kidney disease, and various types of nephropathies. Moreover, oxidative stress seems to at least partly explain the intricate relationship the kidney has with other pathological entities, for instance with cardiovascular comorbidities. Renal replacement therapies give end-stage renal disease patients a fighting chance; however, even these interventions may carry the risk of enhancing existing oxidative stress. Even if nutritional components are not currently routinely used, many have shown promise in preclinical or even clinical studies and could counter some of the deleterious pathways that oxidative stress sets in place. This narrative review provides an update on how these natural nutrients could be beneficial to renal disease patients, and it also aims to give an incentive to future research in the field.

## 1. Introduction

Many natural nutrients have been known for decades, if not centuries. By using modern day experimental methods, the current literature shows they could hold potential for renal disease patients, namely in diminishing the influence or reverting the damage done by oxidative stress, but they can also possess anti-inflammatory, anti-apoptotic, anti-fibrotic, or structure-preserving properties. This narrative review at first provides a brief overview of the pathogenesis of some of the main kidney disorders, diving into the interference of oxidative stress in the disease pathways. Then, the paper offers relevant examples of natural components that recent literature has identified as holding promise in the field. Our review might just be a stepping stone to the inclusion of such components in routine practice, pending future randomized clinical trials that will hold evidence for their broad usage.

## 2. Acute Kidney Injury

### 2.1. Pathogenesis

Acute kidney injury (AKI) may occur as a consequence of prolonged ischemia or exposure to toxins, either exogenic (the most frequently encountered ones in practice being contrast agents and various medications such as aminoglycosides or cisplatin) or endogenic (myoglobin, uric acid etc.) [1].

In ischemic AKI, the production of reactive oxygen species (ROS) contributes to the initiation and maintenance of tubular cell lesions [1]. In the ischemic kidney, adenosine triphosphate (ATP) is broken down into hypoxanthine, which in turn is converted to xanthine by the enzyme xanthine oxidase (XO), with the participation of molecular oxygen (O_2_). During this process, the superoxide radical is generated, the latter being toxic for tubular cells as it induces lipid peroxidation in the cell and organelle membranes (particularly in the mitochondria) [2]. Hypoxic lesions are worsened after perfusion and oxygenation are reestablished (reperfusion/reoxygenation) through ROS and reactive nitrogen species (RNS). ROS are generated in the reoxygenated cells with the contribution of XO and the mitochondria, the latter producing superoxide and H_2_O_2_. H_2_O_2_ has harmful effects that can be local (reacting with membrane proteins and lipids) as well as remote, by activating various pathophysiological pathways that generate inflammation and apoptosis [3]. One of the earliest signals of the ROS-enhanced inflammation is the interferon regulatory factor 1 (IRF1), a transcription factor that increases the production of interferon and chemokines by activating proinflammatory genes [4]. On the other hand, ROS is not only potentially harmful, but could also contribute to an adaptive response, or could interfere in the recovery stage of AKI [1].

The nephrotoxicity of contrast agents is induced through multiple mechanisms, such as the amplification of oxidative stress (OxSt) [5,6,7]. By producing vasoconstriction, contrast agents induce hypoxia in the kidney medulla [8], which can lead to ROS formation, the latter harming cellular membranes and DNA. Hence, there is a vicious cycle during which hypoxia and ROS production support and amplify one another.

OxSt is also one of the mechanisms through which certain medications cause acute tubular necrosis. Direct toxicity induces mitochondrial dysfunction, inhibits lysosomal hydrolases, harms membrane phospholipids, and increases intracellular calcium levels, and, as a result, ROS are generated [9].

Cisplatin accumulates in the tubular cells directly proportional to its dosage [10] and increases OxSt, interfering with antioxidant mechanisms and mitochondrial function. It has chemical affinity for thiols; therefore, it interacts with glutathione (GSH) [10]. The reduction or consecutive inactivation of GSH leads to an intracellular buildup of endogenous ROS. In turn, mitogen-activated protein kinases (MAPK) are activated, as well as p53 and p21, consequently inducing the apoptosis of the tubular cells. ROS favors fibrosis both directly and indirectly, through promoting inflammation, with these processes triggering the formation of more ROS, as well as the production of cytokines and growth factors. ROS production occurs in the microsomes as well, mediated by the enzymes of cytochrome P450 [11]. Moreover, cisplatin increases ROS accumulation through its interference in the mitochondrial respiratory chain [12,13].

Aminoglycoside-induced AKI also has OxSt as one of its key components. Aminoglycosides are reabsorbed by the proximal tubular cells, where they build up within lysosomes until they reach critical levels, causing the breakdown of these organelles, hence the release of lytic enzymes. These enzymes alter membrane phospholipids, damage the mitochondria, induce ROS formation, and finally the cells suffer apoptosis and necrosis [9].

The detrimental consequences of myoglobin reside not just in the obstructive effect produced by its precipitation in the tubular lumen, but a direct toxicity is also involved, first and foremost through heightened OxSt [14]. Free iron derived from heme accelerates ROS generation, such as the hydroxyl radical (·OH) [15] and hydrogen peroxide (H_2_O_2_) [16], followed by lipid peroxidation—here, there is a notable involvement of a redox cycle between two states of iron oxidation, ferric (Fe III) and ferril (Fe IV). The reactivity of the ferril state diminishes in an alkaline environment, which explains the usefulness of alkalinization in preventing myoglobin-induced renal injury [17,18], classically attributed to the prevention of myoglobin precipitation as it keeps the latter in a ionic state. The main target of the peroxidation processes could be the mitochondrial membranes, leading to mitochondrial dysfunction, which in turn may contribute to generating ROS and RNS [19].

### 2.2. Nutrients

Astaxanthin is a carotenoid occurring in several foods (microalgae, fish, shellfish) that was shown to have a protective effect in mercury chloride (HgCl_2_)-induced AKI on an animal model, preventing, on one hand, lipid and protein oxidation, and, on the other hand, the inactivation of superoxide dismutase (SOD) [20]. Its use has cardiovascular and metabolic benefits in humans [21,22]; however, there are no studies demonstrating its effectiveness in patients with kidney disease [21,23]. Moreover, a randomized controlled study failed to show a beneficial effect of astaxanthin on oxidative stress or inflammation in kidney transplant patients [24,25].

On an animal model, sinapinic acid (found in wine, vinegar, and plums) was able to diminish systemic and renal OxSt (by impacting GSH, SOD, and catalase) and inflammation (by countering tumor necrosis factor-alpha, TNF-α, and interleukin 6 [IL-6]) [26].

On another animal model of toxic AKI induced by potassium dichromate (K_2_Cr_2_O_7_), the tocotrienol-rich fraction of palm oil produced a protective effect by increasing GSH, enhancing catalase activity and renal excretion of nitric oxide (NO) and reducing lipid peroxidation [27]. In animals subjected to renal ischemia, γ-tocotrienol balanced renal levels of ATP and preserved mitochondrial function, while also supporting kidney recovery [28].

Phenolic extracts of soybean (*Glycine max*) diminish the nephrotoxicity of cisplatin by decreasing lipid peroxidation, decreasing oxidant enzyme activity (myeloperoxidase [MPO], XO) and the nitrate/nitrite serum ratio, and by boosting the action of antioxidant enzymes (SOD, catalase, glutathione-S-transferase) and increasing GSH levels [29].

Tetramethylpyrazine (ligustrazine), found in natto (a traditional Japanese food made of fermented soybeans), protects the kidney from ischemic/reperfusion lesions by reducing OxSt (it decreases the activity of MPO, the amount of malondialdehyde [MDA], and lipid peroxidation, while heightening SOD activity). It also prevents inflammation and apoptosis [30].

Troxerutin protects the kidney from the toxic effect of D-galactose by increasing the activity of antioxidant enzymes and reducing lipid peroxidation, as well as by blocking the inflammatory response mediated by nuclear factor kB (NF-κB), a transcription factor involved in multiple inflammatory pathways [31].

*Thunbergia laurifolia* leaf extract (a plant used in Thailand) prevents lead nitrate-induced kidney injury by reducing OxSt (it diminishes lipid peroxidation, increases GSH levels, and boosts the activity of the antioxidant enzymes catalase, glutathione reductase, GR, and glutathione peroxidase, GPx). Examples of the active compounds in the leaf extract that could produce these beneficial effects are chlorophyll derivatives, apigenin, and caffeic acid [32].

*Nigella sativa* (black cumin) is rich in thymoquinone (and its derivates), which possesses antioxidant properties (it purges ROS, such as superoxide, hydroxyl radicals, and singlet molecular oxygen) [33]. It also has anti-inflammatory properties. Nigella sativa essential oil has shown antioxidant traits as well [33], particularly directed towards the mitochondria, and it inhibits H_2_O_2_-induced apoptosis [34]. Therefore, Nigella sativa is useful in dampening the ROS-mediated kidney injury that occurs as a result of certain toxins [35], as well as in ischemic-reperfusion injury [36].

Quercetin, a flavonol that is found in many vegetables (onion, cauliflower, cabbage) and fruit (apples, grapes, berries) possesses antioxidant properties [37] (such as lipid peroxidation prevention [38], glutathione preservation [39], and ROS elimination [40]), and diminishes ischemic-reperfusion renal injury and the inflammatory pathways that the latter triggers (it shares this action with curcumin) [41]. It also protects the kidneys from the harmful effects of methotrexate [42] and cisplatin (without reducing its cytostatic efficiency) [43]. However, randomized trials on humans assessing the role of curcumin in contrast-induced nephropathy (CIN) failed to yield statistically significant results. In a randomized double blind placebo-controlled clinical trial, patients given curcumin 24 h before contrast administration as well as up to 48 h after had a lower incidence of CIN (22.7% vs. 32.3% in the placebo group), although the difference was not statistically significant (there was a small number of patients) [44]. The results were similar in patients at high risk of CIN, namely patients with CKD. Given for 5 days (2 days before and 3 days after contrast was administered), curcumin resulted in a statistically insignificantly lower incidence of CIN (16.7% vs. 23.3%) [45].

Resveratrol is a polyphenol with antioxidant actions (it eliminates ROS [46], prevents oxidative lesions of DNA [47], and amplifies the expression of antioxidant enzymes such as SOD and catalase through the Nrf2, nuclear factor (erythroid-derived 2)-like 2, pathway) [48]. Nrf2 is a transcription factor with a key role in cytoprotection. In an oxidative state, it is transported from the cytoplasm (where it is sequestered by Keap1 or Kelch-like ECH-associated protein 1) to the nucleus, leading to the expression of detoxifying and antioxidant enzymes [49,50]. Resveratrol also has antiproliferative and anti-inflammatory properties. Despite its existence in multiple foods (grapes, berries, peanuts), it is difficult to use resveratrol in practice due to its low bioavailability [51]. On the other hand, resveratrol could be active even in low concentrations and may be recirculated [52]. The metabolites of resveratrol could also have relevant biological activity [53,54]. A recent meta-analysis of clinical studies performed with resveratrol has identified a generally positive trend in its effects; however, there are limitations to consider in terms of the small RCTs to this date and the variable dosages and formulations required for achieving clinical efficacy in different diseases [55]. Resveratrol appears to improve renal function in the general human population as well, as shown by a meta-analysis that included 32 studies, which found that resveratrol administration was associated with significantly lower levels of blood urea nitrogen and serum creatinine and significantly higher glomerular filtration rates; however, the authors considered the evidence to be of low certainty [56].

Polyphenols, such as mixed tannins from *Camellia sinensis* extract (green tea), include, first and foremost, epigallocatechin-3-gallate (EGCG) (with the highest activity) [57], but also epicatechin gallate (ECG), epigallocatechin, and epicatechol. Their capacity to chelate metal ions (Cu^2+^ and Fe^3+^) is at the core of their antioxidant properties [58]. Green tea extract improves renal lesions produced by gentamicin by re-establishing the redox balance, reducing oxidant factors and enhancing antioxidant mechanisms [59].

**Figure 1:**
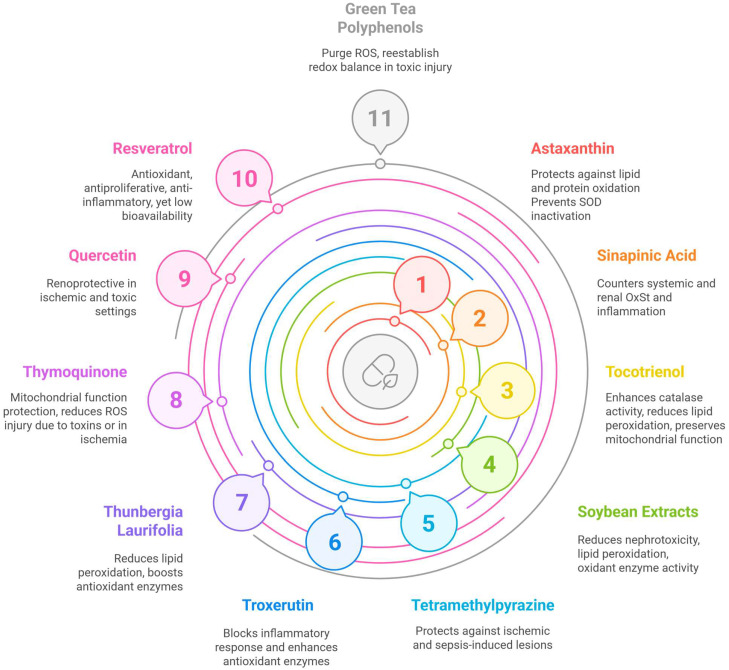
A schematic representation of the natural compounds that may be useful in acute kidney injury. Figure created using Napkin AI Image Generator [https://www.napkin.ai/ (accessed on 27 April 2025)].

An overview of these natural components and their properties is featured in Figure 1. A critical concern is that, in most of these animal model studies, the natural nutrients were administered before the toxic or ischemic exposure [20,27,28,29,30,36,41,42,43] or, in some cases, synchronously [26,32,35,59] and in high doses. Such protocols have very limited applicability and feasibility in humans, and therefore future studies need to consider these severe limitations. The studies that feature antioxidant administration post-insult exposure seem to be rare [31,57].

## 3. Chronic Kidney Disease

### 3.1. Pathogenesis

OxSt plays an important role in the pathogenesis of chronic kidney disease (CKD) as it is involved in the pathophysiology of many of the entities that cause progressive and irreversible renal injury, such as primary glomerulopathies and diabetes [60].

Cellular metabolic changes within CKD promote ROS production, the latter favoring the progression of renal lesions and the appearance and development of CKD complications. A relevant participant is mitochondrial dysfunction that leads to the loss of electrons in the respiratory chain during oxidative phosphorylation, and ROS are consequently generated [61].

The pathways through which OxSt supports the progressive decline in renal function include the following:

- inflammation, apoptosis, and fibrosis [62];
- harming the glomerular filtration barrier [63];
- hypertension (HTN), with a reduction in vasodilators such as NO (through inactivation) and an increase in vasoconstrictors, such as some byproducts of arachidonic acid oxidation [64]. In animals with CKD, an increase in NADH (nicotinamide adenine dinucleotide hydrogen)/NADPH (nicotinamide adenine dinucleotide phosphate) oxidase (NOX) activity and a decrease in SOD action lead to OxSt, which perpetuates HTN and endothelial dysfunction [62].

One of the main factors in the redox imbalance of CKD is excessive superoxide. It is generated by the higher activity of NOX, and improperly eliminated by SOD. NO levels are low due to its increased inactivation by superoxide, which may contribute to HTN, often encountered in CKD patients [62].

The main cause of death in uremic patients is cardiovascular disease, as CKD induces accelerated atherosclerosis and calcification of cardiovascular structures (cardiac valves and arteries). OxSt and inflammation appear to be the link to cardiovascular disease, as both are excessive in CKD. Experiments have shown that in partially nephrectomized animals, inflammation and OxSt markers are increased in the remaining renal parenchyma. There is an increase in lipid peroxidation, GSH is diminished, and NF-κB is activated, as well as monocyte chemoattractants and mononuclear cell infiltrates. NOX and cyclooxygenase-2 (COX-2), as well as 12-lipoxygenase (12-LOX), have a higher activity. Moreover, protective factors (catalase, SOD, GPx, heme oxygenase-1 (HMOX1), NAD(P)H quinone oxidoreductase, and glutamate-cysteine ligase) are inhibited as a result of diminishing the Nrf2-dependent signaling system. Paradoxically, the conditions present in CKD (OxSt, inflammation) are joined by a maladaptive reduction of Nrf2 and a downregulation of its effects, in turn contributing to perpetuating and amplifying kidney dysfunction [65]. An impairment in Nrf2 activity does not strictly affect the kidney and can have deleterious, oxidative, and inflammatory injury-related effects across multiple organs and systems [65,66]. With the positive effects of Nrf2 in mind, it must be noted that high levels of Nrf2 may also have deleterious consequences (including inducing proteinuria by affecting the podocytes [67]). The multifaceted potential of Nrf2 therefore has to be considered when assessing it as a therapeutic target [68,69].

In the context of diminished antioxidant systems, the imbalance between ROS production and clearance contributes to the pathogenesis of cardiovascular complications [70]. As renal function continues to deteriorate, OxSt and inflammation are more and more intense, and protective mechanisms prove faulty. The estimated glomerular filtration rate (eGFR) is directly proportional to antioxidant enzyme activity (SOD and GPx) and inversely proportional to MDA levels. These correlations also apply to inflammatory markers (IL-6 and high-sensitivity C-reactive protein, hs-CRP), which increase the more CKD establishes and progresses. Moreover, renal function deterioration is associated with higher inflammatory and redox markers. Therefore, all these connections suggest a positive feedback loop, a vicious cycle between OxSt, inflammation (activated leukocytes produce ROS), and a decline in renal function, these three components supporting and even amplifying one another, with CKD progressing as a result. There are several consequences, particularly those promoted by inflammation, such as atherosclerosis and cardiovascular disease [71].

Atherosclerosis is also accelerated by the buildup of various components, such as β-2-microglobulin, advanced glycation end products (AGEs), cysteine, and homocysteine, which are victims to perpetuated oxidative aggression. Clearing oxidative products through dialysis diminishes OxSt; however, dialysis also involves the interaction of blood with the extracorporeal circuit (dialyzer membranes with low biocompatibility, long-term intravascular catheters) that may trigger inflammatory and oxidative pathways [72]. Therefore, from this perspective, hemodialysis may constitute a higher risk than peritoneal dialysis [70].

In patients with end-stage renal disease (ESRD) undergoing hemodialysis, antioxidant enzyme activity (SOD, GPx, catalase) is diminished, and markers of lipid peroxidation are increased [73]. Moreover, mitochondrial uncoupling may lead to the release of cytochrome c, which may trigger mononuclear cell apoptosis [74]. Increased cardiovascular morbidity and mortality in these patients is at least partly connected to increased OxSt [75].

ESRD patients undergoing peritoneal dialysis have also increased OxSt paired with a high cardiovascular risk and a faulty ultrafiltration through the peritoneal membrane (harmed by oxidative aggression) with a consequent lower dialysis efficiency [76].

### 3.2. Nutrients

**Figure 2:**
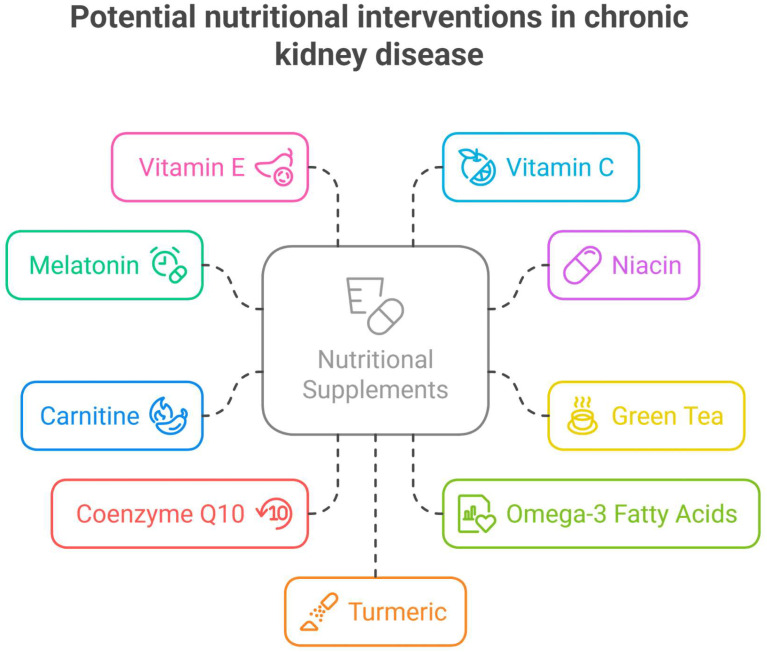
An overview of potential nutritional interventions in chronic kidney disease. Figure created using Napkin AI Image Generator [https://www.napkin.ai/ (accessed on 3 June 2025)].

An overview of the potential nutritional interventions in CKD is provided in Figure 2.

There is a connection between melatonin and the renin-angiotensin-aldosterone system (RAAS) in CKD patients, as its nightly secretion appears to be impaired, leading to circadian dysregulation and renal impairment[77].

In a recent large human cohort of niacin users, it has been found that niacin is associated with a lower mortality and ESRD risk; however, it did bring about a higher risk of CKD, which warrants the need for more clinical trials in the field [78].

When green tea tannins were administered to patients undergoing dialysis, there was a decrease in uremic toxins of low (creatinine), medium (methylguanidine), and large (β_2_-microglobuline) molecular weight. Patients also experienced less joint pain (presumably caused by β_2_-microglobulin joint deposits in the form of amyloid). In the authors’ opinion, this suggests an improvement in the highly oxidative state of dialyzed patients [79]. In a large cohort with an impressive follow-up time (19 years), daily consumption of green tea reduced CKD-related mortality in women, but not in men [80]. Another cohort study identified a dose-dependent tea renoprotection in CKD patients, particularly in the early stages of the disease [81].

Administering carnitine to hemodialyzed patients protects cells from the proinflammatory and pro-oxidative consequences of uremia, since carnitine improves plasmatic antioxidant mechanisms and reduces the activity of JNK (jun-N-terminal kinase), a kinase that stimulates mononuclear cells to produce proinflammatory cytokines when triggered by OxSt [82]. In hemodialyzed children, L-carnitine supplementation reduced IL-6 and fasting blood sugar levels; however, the therapy was assessed only in a short-term and there was a small number of patients included [83]. It has been suggested that carnitine supplementation may be beneficial to anemic, long-term hemodialysis patients [84]; however, currently the evidence is insufficient for a recommendation [85] and the current KDIGO guideline draft also does not recommend carnitine, though it recognizes carnitine supplementation as a cause for hyporesponsiveness to erythropoiesis-stimulating agents [86].

Coenzyme Q10 (CoQ10), also known as ubiquinone, diminishes OxSt in hemodialyzed patients [87]. A meta-analysis has shown that CoQ10 supplementation improves the metabolic profile of CKD patients [88]. In certain trials, CoQ10 reduced inflammation markers in CKD patients [89], although this finding has been previously challenged [90].

In a large meta-analysis, it has been found that markers of seafood-derived omega-3 fatty acids are linked to a lower occurrence of CKD, although the association was modest [91]. When given to ESRD hemodialyzed patients, omega-3 polyunsaturated fatty acids (mainly found in fish oil) reduce OxSt by increasing the activity of antioxidant enzymes (SOD, GPx, catalase); they also diminish lipid peroxidation [92] and block 5-LOX [93]. According to a meta-analysis, omega-3 polyunsaturated fatty acids reduced cardiovascular mortality in hemodialyzed patients; however, it could not be proven that these nutrients could reduce the decline in kidney function or mortality in CKD patients [94].

Vitamin E tends to improve disease progression in children suffering from IgA nephropathy (IgAN), focal segmental glomerulosclerosis (FSGS), and type 1 diabetes, and it improves endothelial dysfunction, lipid peroxidation, and OxSt in CKD patients [95]. In a double-blind, placebo-controlled RCT on vitamin E supplementation in stage 3 CKD patients, it improved renal function, but only up to a threshold of 8 months of usage and there was no measurable impact in the urine/albumin ratio [96]. In a cross-sectional study of over 20,000 patients, vitamin E intake appeared to be a contributor to CKD prevention and it also led to a diminished progression of the disease [97].

In ESRD hemodialyzed patients, vitamin E supplementation increases the activity of antioxidant enzymes (SOD, GPx), reduces lipid peroxidation [73], and diminishes mitochondrial uncoupling and mononuclear cell apoptosis (probably by countering 5-LOX activation and its consequences, such as lipid peroxidation and ROS production) [74]. It also reduces cardiovascular risk, particularly that of myocardial infarction [75]. A meta-analysis has identified lower levels of MDA in hemodialyzed patients with vitamin E supplementation, although there was a significant heterogeneity amongst the included studies, warranting the necessity for further research [98]. Using dialyzer membranes padded with vitamin E reduces inflammatory and oxidative markers [99,100,101] but particularly targets lipid oxidation [102]; it also improves anemia and diminishes resistance to erythropoiesis-stimulating agents [103]. However, such membranes appear to have no impact on other relevant parameters, such as uric acid, albumin, or lipid profile [104].

In ESRD patients that undergo peritoneal dialysis, OxSt can be reduced by the oral administration of a combination of antioxidant vitamins (C and E) [76]. A certain reluctance to the usage of vitamin E has been induced by some meta-analyses that have suggested high doses may increase all-cause mortality [105,106], but this idea has been disproved in other meta-analyses [107,108].

Intravenous administration of vitamin C reduces OxSt markers in ESRD patients under chronic hemodialysis [101]. It appears that vitamin C can positively influence the activity of paraoxonase (an antioxidant enzyme that protects lipoproteins from oxidation) [109]. As per the 2020 update to the KDOQI clinical guidelines, vitamin C supplementation should be considered in CKD patients at risk of having this deficiency [110].

The beneficial effects of turmeric and its ingredient, curcumin, in chronic diseases (including kidney disorders), have been proven in multiple clinical trials, as reviewed elsewhere [111]. In a randomized, double-blind clinical trial on hemodialyzed patients, an 8-week long supplementation with turmeric led to a significant increase in catalase and in albumin levels, and a decrease in MDA [112]. Curcumin reduced NF-kB expression and hs-CRP levels in a another study on hemodialyzed patients; however, there was no proven influence on Nrf2 activity [113]. In an 8-week long trial, a combination of turmeric and Boswellia serrata resulted in a significant decrease in IL-6 (but not in C-reactive protein, TNFα, and GPx) in the treatment group [114]. Curcumin supplementation favorably influenced redox balance in proteinuric CKD patients (as reflected by lower lipid peroxidation in nondiabetics and higher antioxidant capacity in diabetics), albeit without a significant effect on antioxidant enzymes or on the Nrf2 pathway [115]. A combination of curcumin and quercetin also improved early kidney function in patients subjected to renal transplantation, an effect attributed at least partly to HMOX1 induction [116].

It should be noted that exclusively targeting oxidative stress may not be a one-size-fits-all recipe for success in CKD improvement, as a recent meta-analysis has identified that compounds that are not actually antioxidants per se were the most impactful. The authors also point out that the individual levels of oxidative stress should also be evaluated so that a personalized therapeutic formula may be retrieved by future studies [117]. Previous systematic reviews have also identified the need for more trial data to adequately assess the impact of plant-derived compounds on kidney damage [118].

## 4. Renal Senescence

### 4.1. Pathogenesis

OxSt plays a key role in processes related to aging in general [119,120] and particularly in the kidneys, where it contributes to parenchymal deterioration in the elderly. In old animals, glomeruli produce higher quantities of ROS and have increased levels of lipid peroxidation byproducts, and glomerulosclerosis is more pervasive [121]. In senescent kidneys, apart from glomerulosclerosis and a diminished glomerular filtration rate (GFR), there is an increase in F2-isoprostanes (vasoconstrictors produced by lipid peroxidation), in AGEs and their receptor, RAGE, receptor for advanced glycation end-products (their interaction triggering OxSt), and in oxidant-sensitive HMOX [122]. In the distal convoluted tubule, OxSt induced by H_2_O_2_ suppresses the gene for Klotho. Klotho is a transmembrane protein with a β-glucuronidase action. Its reduced production in CKD contributes to CKD-associated degenerative mechanisms similar to aging (arteriosclerosis, osteoporosis, cutaneous atrophy). Klotho gene mutations are generally linked to aging [123]. ROS most likely employ their interaction with the TGF-β1 (transforming growth factor-beta 1) pathway to favor the progression of renal fibrosis as the individual ages [124].

In age-related glomerulosclerosis, podocyte dysfunction also interferes as podocytes are harmed by OxSt and, conversely, protected if OxSt is diminished [125].

Vasodilators (such as bradykinin, enalapril, and amlodipine) suppress oxygen consumption in the renal cortex, which is an effect that appears to be mediated by NO—this has a downfall with age due to a reduction in NO availability, given its consumption by NOX-produced superoxide. Lowered superoxide levels (through clearance or diminished production by NOX inhibition) may restore the vasodilator capacity of reducing oxygen consumption [126].

### 4.2. Nutrients

A vitamin E-rich diet may reduce HMOX-1, F2-isoprostanes, and RAGE levels. It may also counteract glomerulosclerosis, and preserve glomerular perfusion and renal function [122].

Resveratrol ameliorated age-related renal injury in mice by regulating Nrf2 signaling, which in turn led to better renal function and improved proteinuria via decreasing OxSt [127]. Targeting Nrf2 appears relevant in aging kidney research, as its expression declines through time and its impairment affects recovery post-ischemic reperfusion injuries [128]. Nrf2 constitutes a cellular defense mechanism against OxSt and it is an indicator of redox equilibrium in the aging kidney; therefore, maintaining its activity appears critical [127].

Taurine (2-aminoethanesulfonic acid) has antioxidant effects and attenuates age-related glomerulosclerosis. This suggests a link between oxidation and aging, most likely explained through the TGF-β1 pathway, as taurine suppresses TGF-β1 and blocks the TGF-β1-induced synthesis of mesangial collagen [124]. Through an epidemiological study, taurine has been identified as related to longevity [129].

Aging is associated with the systemic (and renal) increase in MAPK activity (extracellular signal-regulated kinase [ERK], JNK, and p38-MAPK), one of the pathways through which OxSt exerts its deleterious effects. This may be reversed by caloric restriction, probably through its antioxidant benefits [130]. Caloric restriction is also involved in preventing lipid peroxidation in mitochondrial membranes, while also diminishing apoptosis [131]. Protein and calorie restriction preoperatively has had positive impact on the renal function of kidney donors [132].

Natural iron chelators may be considered in future research regarding the aging kidney, given that there could be a contribution of iron metabolism to age-related renal dysfunction. In aged rats, while there was a proven iron buildup in the renal cortex, treatment with deferoxamine had limited effects [133].

The flavanol with the highest concentration in cocoa, (−)-epicatechin, notably impacted OxSt markers in the brain, heart, and kidneys of senescent mice, causing a restoration of systemic antioxidant capacities. There was a complete recovery of the ratio between reduced/oxidized glutathione, as well as an improvement in protein carbonyls [134]. Positive changes in MDA and protein carbonyls due to cocoa flavanols were also identified in a randomized controlled trial [135]. Cocoa flavanols may support cardiovascular health as the individual ages [136,137].

## 5. The Kidney and Obesity

### 5.1. Pathogenesis

OxSt is one of the pathways through which obesity promotes glomerulosclerosis, with inflammation and HTN and RAAS activation being amongst the mediators [138]. This has been demonstrated by an increase in inflammation and OxSt markers in obese, overfed animals [138]. Obesity not only contributes to chronic deterioration in the renal function and structure, but also to acute decline, the most important probable participant being OxSt through F2-isoprostanes. Other participants may also be the proinflammatory, pro-oxidative environment and endothelial dysfunction induced by obesity [139]. Sirtuin 1 may also be involved in the appearance of obesity-induced renal damage [140].

### 5.2. Nutrients

Magnolia extract attenuates inflammatory and OxSt markers, while also counteracting the proteinuria and renal structural changes linked to hypercaloric diet-induced obesity [138].

Capsaicin is an activator of the transient receptor potential vanilloid channel 1 (TRPV1), the latter being expressed in adipocytes as well as the kidneys, hence being a potential link between obesity and kidney disease [141,142]. In animal models, capsaicin could lower blood pressure and increase NO production, improving endothelial function [143].

A resveratrol analog, piceatannol (preferable due to its superior solubility and bioavailability), seems to prevent renal lesions induced by obesity, counterbalancing peroxidation and fibrosis mechanisms [144].

An anthocyanin-rich fraction from a traditional type of rice from Thailand, also termed black rice or *Oryza sativa* L. variety “Luem Pua”, was able to improve renal morphology, upregulate Nrf2, HMOX1 and GSH, and diminish MDA; therefore, it attenuated OxSt and kidney cell apoptosis. In high-fat diet animal models, Keap1 expression is increased—this event is known to impede the nuclear translocation of Nrf2 and therefore prevents its potential antioxidant and detoxifying effects [145].

The ability to assist in weight management has also been demonstrated in human trials for Magnolia extract combined with *Phellodendron amurense* [146], for capsaicin [147], for *Cyperus rotundus* extract containing piceatannol [148], and for black rice extract [149]; however, these trials have not explored the potential beneficial effects on kidney function.

## 6. Kidney and Cardiovascular Disease

### 6.1. Pathogenesis

OxSt, inflammation, and HTN amplify one another in a relationship that is deleterious to the body and, particularly, to the kidney and the cardiovascular system. Inflammatory cell infiltrates within the renal interstitium produce ROS and can be involved, alongside NOX activation and through OxSt, in the pathogenesis of at least some forms of HTN. From the opposite point of view, on animal models of HTN, antioxidants improve HTN as well as tubulointerstitial inflammation and NF-κB activation [62].

Aside from its vasoconstrictor properties, as well as the sodium retention it induces (directly by increasing the activity of the sodium-hydrogen antiporter in the proximal convoluted tubule, and indirectly by stimulating the adrenal glands to produce aldosterone), angiotensin II (AT2) has deleterious consequences mostly mediated by an increase in OxSt (endothelial dysfunction, promoting fibrosis and inflammation) [150], potentially producing renal, cardiac, and vascular injury. AT2-induced OxSt is probably involved in the pathogenesis of renovascular HTN, an idea supported by the increased expression of the AT2 receptor and NOX in the ischemic kidney [151]. RAAS involvement in OxSt amplification is proven by the antioxidant action of angiotensin-converting enzyme inhibitors (ACE inhibitors), adding to their preventive effects on endothelial dysfunction [152]. ACE inhibitors (captopril, enalapril) enhance the activity of antioxidant enzymes (CuZn-SOD, Mn-SOD, and Se-GPx, but not catalase), which suggests these medications possess a protective effect against OxSt [153].

Through the generated superoxide, NOX plays an essential role in the proliferation of vascular smooth muscle cells. AT2-induced hypertrophy of the vascular smooth muscle is exerted through NOX amplification, and therefore through an increased production of superoxide. Conversely, blocking NOX reduces the hypertrophy of arteriolar muscle cells. [154].

By interacting with the mineralocorticoid receptor in the arteriolar wall, aldosterone produces remodeling hyperplasia (on the smooth muscle and extracellular matrix); it also increases OxSt, leading to endothelial dysfunction, repressing vasodilation, and inducing fibrotic and inflammatory phenomena [155].

The cardiorenal syndrome entails a bidirectional amplification of cardiac and renal dysfunction, which can cause a vicious cycle leading to progressively worsening morphologic and functional consequences in both these organs. This syndrome may be induced by excessive ROS production resulting from an imbalance between RAAS, the sympathetic nervous system, and inflammation [156].

### 6.2. Nutrients

Resveratrol reduces the mitochondrial dysfunction induced by aldosterone [157]. It also reduces blood pressure, body mass index, and lipid profiles [158]. Moreover, it improves left ventricular systolic and diastolic function, global longitudinal strain, and quality of life in some RCTs [55]. A randomized, double-blind, placebo-controlled study on patients suffering from CKD and diabetes showed that resveratrol supplementation may mitigate the cardiovascular risk profile of these subjects by independently and positively influencing endothelial function [159].

Vitamin C, probably through its antioxidant effect, represses the AT2 receptor in the ischemic kidney and enhances GPx activity [151]. In a meta-analysis, however, vitamin C did not seem to have significant impact on cardiovascular disease or events in general [160].

CoQ10 supplementation induces a dose-dependent systolic blood pressure reduction, especially with long-term usage [161]. This effect is most likely mediated by an improvement in endothelial function [162], but also CoQ10 may interfere with RAAS and decrease the level of aldosterone [163]. A meta-analysis of human studies has shown that CoQ10 supplementation is associated with a significant improvement in endothelial function, which may be relevant for cardiovascular [164] and kidney protection.

Curcumin (a polyphenol, diferuloylmethane, giving turmeric its yellow color) can have potent cardiovascular protective effects through a variety of mechanisms. It lowers the vasoconstriction induced by AT2, counters the activity of various cell adhesion molecules, has antioxidant and cytoprotective effects, and scavenges ROS [165]. Curcumin appears to have beneficial cardiovascular effects even in nanorange formulations, as shown by a meta-analysis. It significantly improved lipid profile, and reduced blood pressure as well as inflammation [166], which might result in a better preservation of kidney function.

## 7. Diabetic Nephropathy

### 7.1. Pathogenesis

The various pathophysiological pathways that lead to diabetic nephropathy (DN) all have OxSt as one of their main mediators [60]. OxSt triggers inflammatory mechanisms that heavily contribute to the start and progression of the complications of diabetes. OxSt also perpetuates the progression of these complications by enabling glomerulosclerosis [167]. OxSt is brought about by hyperglycemia [168].

Hyperglycemia interferes in multiple regulatory pathways that amplify OxSt; conversely, hyperglycemia-induced OxSt may also disturb these pathways [169]. These regulatory pathways include protein kinase C (PKC) with its various isoforms (PKCα, PKCβ, PKCε), and MAPK, such as p38, JNK şi ERK1/2, NF-κB, and TGF-β1 [170,171]. Mitochondria-dependent apoptosis is induced through multiple mechanisms, such as the following: (1) TNFα release that, by activating caspase-8, leads to the cleavage of Bid (BH3 interacting-domain death agonist) to truncated Bid, tBid (a pro-apoptotic protein); (2) caspase-9 and caspase-3 activation, which induce the cleavage of PARP [poly(ADP-ribose)polymerase]; (3) an altered balance between proapoptotic and antiapoptotic influences (Bax and Bcl-2 also known as bcl-2-like protein 4 and B-cell lymphoma/leukemia 2 protein) [171].

The production of AGEs and protein carbonylation heavily contributes to the appearance of OxSt-induced glomerular lesions in DN [172]. By interacting with RAGE, AGEs generate OxSt, leading to inflammation and vascular thrombosis [173].

### 7.2. Nutrients

Curcumin diminished lipid accumulation and OxSt in the kidneys of an animal model of DN, therefore having a nephroprotective effect mediated by the AMPK signaling pathway (adenosine monophosphate-activated protein kinase) and by Nrf2 [174,175]. Curcumin appears to activate the Nrf2/antioxidant response element (Nrf2/ARE) pathway and its cytoprotective gene expression via protein kinase C [175]. Curcumin induces the expression of antioxidant enzymes such as SOD and catalase [176], GR and GPx [177], HMOX1 (via Nrf2 activation) [174,178], glutathione S-transferase, NAD(P)H quinone oxidoreductase 1 [179], and γ-glutamylcysteine ligase [165,175]. In a randomized, placebo-controlled study performed on patients suffering from DN, turmeric supplementation for 2 months led to reduced proteinuria and significantly decreased serum TGF-β as well as serum and urinary IL-8 (both of which are pathogenetic contributors to DN), but did not improve creatinine levels (albeit the study was short term), nor did it alter TNF-α [180].

Diosmin improves metabolic parameters (glycemia, insulinemia, body weight) and positively influences redox factors (MDA, SOD, catalase, GSH, NO) and morphologic indicators (it rebuilds the renal architecture), once NF-κB is normalized [170]. In vitro studies have shown that diosmin can counter hyperglycemia-induced endoplasmic reticulum stress in proximal tubular cells [181].

Mangiferin, a xanthone with a polyhydroxy-polyphenolic structure, prevents apoptosis by blocking OxSt-activated proapoptotic mechanisms and their corresponding signaling pathways (mediated by proteinkinases, NF-κB, TGF-β1) [171]. In an animal model, mangiferin reduced DN progression by countering mesangial matrix expansion [182].

*Bacopa monnieri* (also named brahmi in ayurvedic practice, where it is used mostly as a nootropic) and stigmasterol (one of its main active components) attenuate the biochemical disturbances related to DN (glycemia, nitrogen retention, lipid profile) and also prevent DN progression by supplying protection against OxSt (through SOD and GSH) and by reducing lipid peroxidation and AGE production [183].

Oleanolic acid (a bioactive principle with anti-inflammatory, hypolipidemic, and antioxidant effects, occurring in multiple fruits and vegetables) and N-acetylcysteine (involved in GSH regeneration) increase insulin secretion and SOD levels and reduce triglycerides and albuminuria. They also favor kidney tissue recovery by increasing nephrin, increasing endothelial cell-selective adhesion molecules, inhibiting the TGF-β/Smad pathway, and reducing the stress on the endoplasmic reticulum [184].

Resveratrol may prevent DN by reducing intracellular ROS levels (via NOX inhibition [185] through the JNK/NF-κB pathway [168]). This halts the transition of the tubular epithelium to a mesenchymal phenotype by reducing the activity of ERK1/2 [185]. Resveratrol also balances mesangial proliferation and fibronectin expression [168]. On the other hand, a meta-analysis found no evidence of resveratrol affecting renal function in patients suffering from diabetes and on hypoglycemic medication [186], whereas a later RCT contradicted this conclusion in elderly diabetic patients [187].

Through its antioxidant effect, EGCG (one of the most valuable polyphenolic tannins from green tea) attenuates the renal lesions that occur in diabetes, and reduces proteinuria, lipid peroxidation, and the accumulation of AGEs [188]. In rats with type 1 diabetes, green tea administration prevented DN worsening independently of glycemia levels [189]. When green tea polyphenols were given to patients maximally treated with RAAS inhibitors, residual albuminuria was reduced, an effect attributed to the ability of these natural compounds to diminish podocyte apoptosis [190].

The mitochondrial deficit of oxidized CoQ10 may be one of the factors that induce DN; therefore, ubiquinone supplementation supports mitochondrial function and prevents DN [191]. On animal models of DN, CoQ10 reduced oxygen consumption by preventing the functional uncoupling and morphologic fragmentation of mitochondria; it also diminishes glomerular hyperfiltration and proteinuria [192]. CoQ10 therefore appears able to reset the mitochondrial dysfunction brought about by DN [193]. CoQ10 may improve the glycemic control and, partly, the lipid profile of DN patients, but the evidence so far is scarce according to a systematic review and meta-analysis [194].

α-Lipoic acid reduces OxSt in the diabetic kidney (it diminishes MDA and F_2_-isoprostanes while increasing GSH), which is proven by a slowed antioxidant enzyme activity [195,196] and is associated with a reduced expansion of the mesangial matrix and diminished glomerulosclerosis [197]. In a meta-analysis of randomized controlled trials on human subjects, a combination of valsartan and lipoic acid increased SOD levels and decreased MDA, countering OxSt and lowering albuminuria in DN patients more significantly than either of the components in monotherapy [198].

In a non-randomized human study, in ESRD diabetic patients treated with a combination of green tea extract (mainly EGCG) and Indian gooseberry (*Emblica officinalis*), an antioxidant effect was exhibited with the inhibition of AGE formation and an improvement in the plasmatic redox balance[199].

## 8. Glomerulopathies

### 8.1. Pathogenesis

OxSt is involved in the pathogenesis of multiple glomerulopathies (GP), such as minimal change disease (MCD), membranous glomerulopathy/nephropathy (MN), FSGS, IgAN, lupus nephritis, the controversial IgM nephropathy, and post-streptococcal glomerulonephritis, as well as rapidly progressive glomerulonephritis and anti-glomerular basement membrane (GBM) disease [60,200,201].

In FSGS and MN, apart from the characteristic lesions, there is also a progressive podocyte dysfunction linked to an increased expression of TGF-β in podocytes. Its triggers are the mechanical pressure and biomechanical overload of the podocytes, which induce higher TGF-β and AT2 levels. OxSt increases due to AT2 and activates TGF-β, thus triggering glomerulosclerosis. Glomerulosclerosis firstly induces the thickening of the GBM (with an increased production and low clearance of GBM structural proteins), associated with podocyte detachment from the GBM (potentially followed by their apoptosis or their transformation into mesenchymal cells, which in turn promotes glomerulosclerosis)—mechanisms supported by high levels of TGF-β. TGF-β produces its effects by triggering the Smads and MAPK/ERK pathways within podocytes. With time, during glomerulosclerosis, there is an expansion of the mesangial matrix, most likely through a podocyte-induced paracrine stimulation of the mesangial cells, mediated by the conjunctive tissue growth factor and the vascular endothelial growth factor. The production of these growth factors is induced by TGF-β through the Smad pathway [202,203].

In MN, OxSt also interferes apart from the immune system [204]. In Heymann nephritis, an animal model of MN, OxSt is a crucial pathogenic factor, with the GBM containing ROS. Lipid peroxidation produces highly reactive aldehydes (such as MDA and 4-hydroxynonenal) that react with the lysine residues of structural proteins, forming adduction products. These products have been identified in the glomeruli of animals suffering from Heymann nephritis. ROS can alter GBM proteins directly by unraveling peptide bonds, as well as through a chemical reaction between H_2_O_2_ and a halogen (such as Cl^−^) catalyzed by MPO [205]. OxSt’s role in the pathogenesis of MN is also supported by HMOX1’s ability to counter pathogenesis through its antioxidant effects, while it also prevents apoptosis and induces immune modulation [206]. It has also been proven that OxSt interferes in the pathogenesis of IgG4-related MN, as the latter’s pathophysiology includes intracellular acidification, mitochondrial dysfunction, ROS generation, and disruption in the podocyte cytoskeleton [207]. Aldose reductase and SOD2 could constitute antigens that are immunologically targeted in MN. SOD2 glomerular expression is increased under the influence of OxSt [208].

The imbalance between ROS and antioxidant mechanisms takes part in the pathogenesis of MCD [209]. The intensity of lipid peroxidation (shown by MDA) is correlated to the recurrence rate of MCD: the former is greater in patients having a recurrent attack as opposed to those in remission. Lipid peroxidation also decreases if the patient is about to enter a remission phase [200,210]. An animal model of MCD is adriamycin-induced nephropathy, in which the podocyte foot processes are effaced and there is a nephrotic syndrome. The role of OxSt in the appearance of this nephropathy is supported by two main elements: an iron chelator, dexrazoxane, can prevent MCD (it inhibits the formation of hydroxyl radicals) [211] and there is a diminished reductive capacity of the renal mitochondria (after adriamycin administration and before proteinuria ensues) [212]. OxSt also interferes in puromycin aminonucleoside nephrosis (or PAN, another model of MCD) [213], in which H_2_O_2_ is generated in the GBM, with CYP2B1 (a major isoform of cytochrome P450) playing an essential role [214,215] as well as, potentially, podocyte NOX [216].

Possibly linked to mitochondrial dysfunction, the involvement of OxSt in the occurrence of FSGS [217] is proven by the increase in serum and urinary MDA, as well as by the high levels of MDA and SOD in the glomeruli. Glomerular MDA correlates to the degree of glomerulosclerosis [218].

In IgAN, there are characteristic IgA1 molecules that possess galactose-deficient O-glycans in the hinge region (termed aberrantly glycosylated IgA1 or Gd-IgA1) that can act as epitopes for anti-glycan antibodies (IgG or IgA1). The consecutive immune reaction triggers ROS production within mesangial cells, which has been demonstrated by an increase in lipid peroxide and MDA in the serum and erythrocytes of IgAN patients, as well as by a diminished activity of antioxidant enzymes (SOD, catalase, GPx) [219]. Moreover, advanced oxidation protein products (AOPPs), a marker of OxSt, are increased in the serum of IgAN patients and are associated with Gd-IgA1 levels, as well as with disease progression (entailing proteinuria and renal dysfunction). This demonstrates that the disease features and its progression are linked to the intensity of OxSt [219,220,221]. Oftentimes transported by albumin, AOPPs increase OxSt in the blood, as a vicious cycle is created: OxSt generate AOPPs, which in turn amplify OxSt. Systemic OxSt enhances the nephrotoxicity of Gd-IgA1, which supports the idea that OxSt triggers and modulates IgAN progression [219,221]. Other arguments in favor of OxSt’s involvement in the pathogenesis of IgAN are as follows: increased levels of OxSt markers; diminished antioxidant mechanisms within the erythrocytes of IgAN patients; the susceptibility of low-density lipoproteins (LDL) to oxidation; the increase of AGEs in patients with renal dysfunction; the higher plasmatic markers of lipid peroxidation [222].

In IgAN as well as in non-IgA mesangial proliferative glomerulonephritis, the decrease in SOD activity (a cause and/or consequence of the pathophysiological mechanisms involved) increases the vulnerability to OxSt due to the insufficient clearance of superoxide [223].

There is a redox imbalance in patients with lupus nephritis as OxSt increases and antioxidant potential plummets [224]. An adequate antioxidant activity that effectively counters OxSt may explain why the disease remains inactive in some patients [225]. The Nrf2 pathway may dampen disease manifestations by reducing OxSt and blocking inflammatory (inhibiting NF-κB) and fibrotic (downregulating TGF-β1) mechanisms; Nrf2 also scavenges ROS [226]. Among various biomarkers of the increase in OxSt in systemic lupus erythematosus patients, serum thiols are indirectly proportional to disease activity levels as well as to the degree of renal dysfunction, ably differentiating patients with or without renal injury [227].

### 8.2. Nutrients

Resveratrol could be beneficial in MN given that, in an animal model of the disease, it reduces glomerular injuries and proteinuria by heightening HMOX1 activity (possibly through the Nrf2 pathway by stimulating Nrf2 binding within podocytes). Resveratrol may also hinder ROS production, counter apoptosis, and complement activation [228].

Apocynin (also termed acetovanillone, an organic compound structurally related to vanillin) reduces proteinuria in animals suffering from PAN by inhibiting NOX and therefore counteracting superoxide production in podocytes, as well as by countering FcRn-mediated albumin transport (FcRn or the neonatal fragment crystallizable [Fc] receptor plays a role in albumin turnover) [216]. Apocynin was first isolated from Apocynum cannabinum, a plant used in phytotherapy as well as in homeopathy for treating cardiac or renal hydrosaline overload (edema, ascites, hydrothorax, anasarca).

By countering OxSt markers (renal MDA) and fibrotic cytokines (TGF-β1), α-tocopherol can diminish IgAN-associated proteinuria [229,230]. Unfortunately, recent data are lacking in this domain, and, moreover, a recent analysis found no significant association between vitamin E and IgAN or MN [231].

Nephrokeli, a Chinese herbal formula, can reduce proteinuria and renal lesions in IgAN patients by diminishing OxSt, inflammation, and fibrosis markers [232].

In a randomized, placebo-controlled study on a small number of patients suffering from lupus nephritis, supplementation with turmeric for 3 months decreased hematuria, proteinuria, and systolic blood pressure; however, there was no proven impact on the immunological basis of the disease [233].

## 9. Nephrolithiasis and Obstructive Nephropathies

### 9.1. Pathogenesis

If the link between nephrolithiasis and CKD appears self-explanatory, the connection of the former to cardiovascular (including HTN) and metabolic diseases (diabetes, metabolic syndrome) may appear surprising, if OxSt is disregarded as an important mediator. Oxalocalcic (the most common form of nephrolithiasis) and calcium phosphate lithiasis are both linked to higher ROS production, as cell culture and animal model studies have proven ROS generation is a component of the interaction of these stones to the renal epithelium. These experimental studies correlated to clinical studies have shown the increase of OxSt in patients suffering from nephrolithiasis [234].

OxSt also interferes in the pathogenesis of renal lesions induced by ureteral obstruction (UrOb) [235], in which ischemia, inflammation, and apoptosis play a key role [236]. OxSt is increased in the faulty as well as in the contralateral kidney [237]. UrOb leads to the following: suppression of antioxidant enzymes [237,238,239,240]; more ROS (particularly hydroxyl and superoxide radicals) [239]; RNS and AGE production [241]; lipid peroxidation [237,240,242]; and oxidized protein degradation [241,243] as well as DNA degradation [244]. UrOb can also bring about enhanced NOX [240] and HMOX1 activity [243], as well as a higher expression of reaction molecules such as heat shock proteins [237].

XO plays a role in the occurrence of OxSt in the UrOb affected kidney, which has been demonstrated by the way that febuxostat, through inhibiting XO, diminishes OxSt, inflammation (cytokine secretion, macrophage infiltrates), and fibrosis (the production of TGF-β1, α-SMA [alpha smooth muscle actin, a marker of myofibroblastic proliferation], and type 1 collagen) [245].

In congenital obstructive nephropathy, renal injuries progress through apoptosis (modulated by OxSt and inflammation), followed by fibrosis (including the accumulation of extracellular matrix). The mediators that govern these mechanisms are the cytokines and growth factors released from the tubular cells that have been injured by interstitial macrophages and myofibroblasts. A key role is held by AT2, which acts as a hormone with pro-oxidative (increasing OxSt especially in the mitochondria), proinflammatory (inducing cytokine and chemokine production, NF-κB activation, and adhesion molecule expression), and fibrotic (triggering growth factor production, especially TGF-β1) properties. By amplifying OxSt and inflammation, AT2 can induce apoptosis, as well as consecutive fibrosis; therefore, AT2 is an essential regulator of renal fibrogenesis [150], which is why agents that block its production [239] or action [240] have a preventive role.

Aldosterone also promotes OxSt, inflammation and fibrosis, as is demonstrated by the ability of eplerenone, a selective antialdosteronic agent, to inhibit these processes in the UrOb-affected kidney [246].

### 9.2. Nutrients

Nutrient therapies in these diseases should be regarded as a potential adjuvant step, pending future research, as it is a well-known fact that the primary therapeutic goal is relieving the obstruction [247].

Quercetin reduces UrOb-induced inflammation and apoptosis [236]. Quercetin proved renoprotective effects in animal models of obstructive nephropathy through antifibrotic mechanisms (via β-catenin yet independently of Smad3 - mothers against decapentaplegic homolog 3) [248].

Resveratrol inhibits UrOb-triggered interstitial renal fibrosis through its antioxidant properties and by influencing the TGF-β1/Smad3 pathway [249,250,251].

EGCG, the dominant and most active tannin in green tea, protects the kidney from UrOb-related inflammatory and oxidative aggression and also prevents the consequent interstitial fibrosis. It influences the nuclear translocation of two transcription factors, NF-κB and Nrf2 (producing a higher Nrf2 nuclear accumulation in renal tubular epithelial cells, potentially acting to compensate the inflammation initiated by NF-κB. It increases the activity of antioxidant enzymes, enhances the enzymes involved in producing antioxidant components [57,252], and it inhibits the TGF-β/Smad pathway [253].

Tocopherol (vitamin E) could dampen oxidative and cytotoxic mechanisms triggered in the UrOb-injured kidney, for instance reducing LDL oxidation [254], although it did not prove its efficiency in countering consequent fibrosis [244] and the literature lacks recent data.

Lecithin counters tubulointerstitial lesions in the UrOb-affected kidney through complex means that include a reduction in OxSt, inflammation (by diminishing leukocyte infiltration and NF-κB pathway activation, as well as producing cytokine), and fibrosis (it reduces TGF-β1 and α-SMA levels) [255].

Melatonin reduces UrOb-related renal injury by counteracting OxSt and the inflammatory and apoptotic reactions the latter induces (it decreases the activation of NF-κB and p38-MAPK) [256]. It also inhibits the suppression of renal aquaporins, has anti-inflammatory effects, and preserves kidney morphology after UrOb [257].

In a small, randomized clinical trial, the supplementation with Nigella sativa was proven to result in the disappearance or shrinkage of kidney stones [258].

## 10. Discussion

Although these nutraceuticals appear to have pleiotropic effects, it must be kept in mind that, currently, available data from human studies are either limited or do not support a beneficial effect of these compounds. More trial data (on human subjects in particular) are needed to define the usefulness of these compounds in preventing kidney damage [118].

For instance, astaxanthin has been the subject of multiple human clinical trials [21], and, while very few studies have addressed renal disease, cardiovascular and metabolic benefits have been shown [22], as well as its ability to mitigate inflammation and oxidation [23], however, it had no proven impact on oxidative stress in patients who have undergone renal transplantation [24,25]. Although relatively short-term studies seem to indicate a positive effect on oxidative stress, this effect does not seem to be maintained with long-term administration, as indicated by the results of a 12-month prospective study on kidney transplant patients [21].

The human trials on curcumin have shown a positive impact in patients with CKD, ESRD, DN, or lupus nephritis [111,180,233]. Curcumin might also prevent CIN, although the available studies failed to reach statistically significant results, possibly due to their low power [44,45]—larger studies are clearly needed.

From a clinical point of view, it is important whether targeting oxidative stress and/or inflammation brings about palpable changes in terms of improved renal function: Do biochemical benefits translate into clinical benefits? For instance, in a small, randomized, double-blinded, placebo-controlled trial on CKD patients, curcumin and Boswellia serrata reduced IL-6 levels, proving that these compounds can target inflammation in CKD. While there was no difference noted between groups for creatinine levels, the follow-up time was short. Longer follow-up times are required in future studies on plant nutrients to reliably observe their impact on kidney function [114].

Another important question is regarding the pharmacokinetics of these nutraceuticals: Are their metabolites relevant? Is there recirculation, and if so, what is its clinical impact? An example is resveratrol, one of the more thoroughly studied natural compounds in humans. Despite its low bioavailability [51], it seems that its benefits may be preserved due to the biologically active potential of its metabolites [53,54]. In vitro, it has been demonstrated that resveratrol may be active even in very low concentrations. Moreover, resveratrol may be recirculated, with a secondary peak in its concentration 6 h after ingestion [52].

The dose dependence of the effects is another important issue, clearly illustrated by resveratrol. In a meta-analysis on the renal effects of resveratrol in the general population, the dose needed to reduce blood urea nitrogen levels (<500 mg daily) was lower than the dose required for an impact on creatinine levels (>1000 mg daily). The reasons for this discrepancy are worth exploring [56]. However, a previous meta-analysis found no evidence that resveratrol improves renal function in patients suffering from diabetes and on hypoglycemic medication, though there were positive results regarding blood pressure and blood glucose control [186]. In a later RCT, however, 500 mg/day of resveratrol given for 6 months had a positive impact on renal function in elderly diabetic patients [187]. High-quality evidence is needed to establish an adequate, personalized approach for the desired beneficial effects to be achieved.

**Figure 3:**
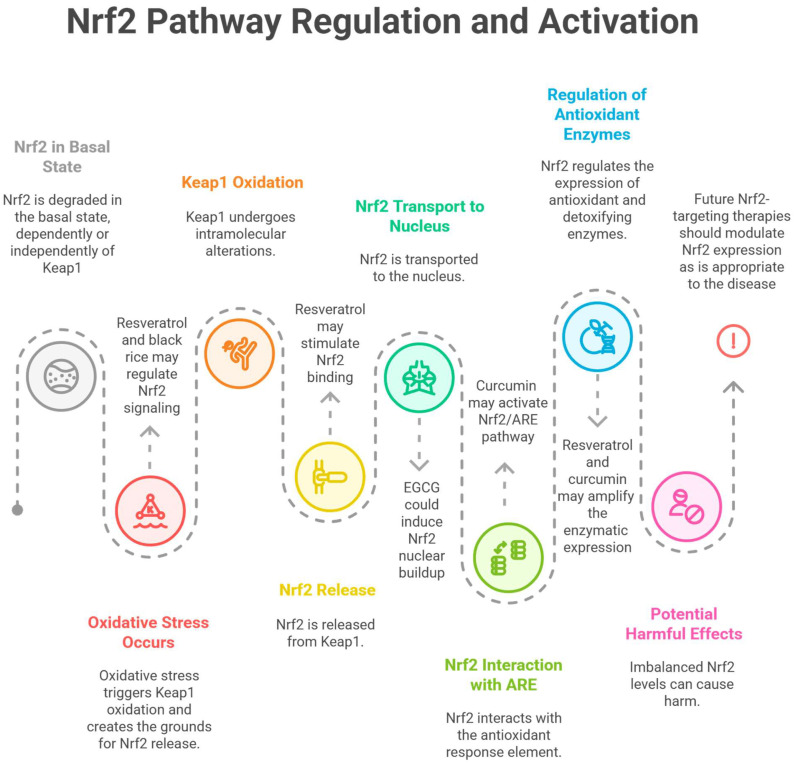
A schematic representation of the Nrf2 pathway, its regulation and activation, and how some natural antioxidants may interfere. Figure created using Napkin AI Image Generator [https://www.napkin.ai/ (accessed on 3 June 2025)].

Finally, whereas targeting Nrf2 with various nutraceuticals seems appealing, a balance must be struck as both high and low levels of NRF2 may have multiorgan deleterious effects [65,66,68]. Nrf2 is a protein with oxidative stress-modulating properties, negatively regulated partly by Keap1. Nrf2 is degraded by the proteasome, either Keap1-independently (involving F-box/WD repeat-containing protein 1A and glycogen synthase kinase 3) or Keap1-dependently (via the E3 ubiquitin ligase). AMPK as well as MAPK may also interfere with the activity of Nrf2. In the basal state, Nrf2 degradation is stimulated; however, in an oxidative state, Keap1 is oxidized, leading to the release and consequent buildup of Nrf2, allowing Nrf2 to be transported to the nucleus, where it regulates multiple antioxidant and detoxifying enzymes by interacting with the DNA sequence known as the ARE [49,50,69]. Nrf2 induction may aggravate proteinuria by harming podocytes, thereby worsening kidney impairment [67]. Therefore, modulating the expression of Nrf2 is one of the nephroprotective mechanisms of herbal products [69]. The Nrf2 pathway and the potential points of impact of natural antioxidants are summarized in Figure 3.

## 11. Conclusions

Most positive evidence regarding the beneficial consequences of the antioxidant effects of natural products in renal disorders is derived from animal studies. By contrast, the evidence provided by human studies is either weak or contradictory (or both). There are, however, glimmers of hope for several phytocompounds (each of them marred by shortcomings), including the following: resveratrol and/or its analogs, which have multifaceted properties (anti-inflammatory, antioxidant, antifibrotic), though there are uncertainties regarding the optimal dose and limitations engendered by the reduced biovailability, hence the need to identify the appropriately bioavailable formulation; green tea polyphenols, which have been proven to be endowed with antioxidant, function-preserving, anti-inflammatory, and antifibrotic properties, though they do not seem to be equally effective in both sexes; and vitamin E equivalents, which have antioxidant, antiapoptotic, and antifibrotic properties, though their effects in many renal diseases have scarcely been explored.

Research objectives in AKI differ from those appropriate for CKD. Regarding acute kidney injury, the evidence on natural compounds heavily relies on animal studies, in which these compounds are usually given in high doses and before the insult occurs, hence their limited applicability in human disease. Therefore, future research should focus on preventing various forms of AKI because there is no proven effective conventional treatment—it is worth studying their preventive ability in circumstances where the occurrence of AKI is highly likely, such as cardiac surgery or the administration of contrast agents in high-risk patients.

As for CKD, although conventional medicine has made impressive strides in the treatment thereof, there are ample opportunities for intervention for natural products, especially on the mechanisms of progression which are only marginally influenced by conventional treatment, such as inflammation and oxidative stress.

## Abbreviations

The following abbreviations are used in this manuscript:

α-SMA	alpha smooth muscle actin
ACE	angiotensin-converting enzyme
AGEs	advanced glycation end products
AKI	acute kidney injury
AMPK	adenosine monophosphate-activated protein kinase
AOPPs	advanced oxidation protein products
ARE	antioxidant response element
AT2	angiotensin II
ATP	adenosine triphosphate
Bax	bcl-2-like protein 4
Bcl-2	B-cell lymphoma/leukemia 2 protein
Bid	BH3 interacting-domain death agonist
CKD	chronic kidney disease
CIN	contrast-induced nephropathy
CoQ10	coenzyme Q10
COX-2	cyclooxygenase-2
DN	diabetic nephropathy
ECG	epicatechin gallate
EGCG	epigallocatechin-3-gallate
eGFR	estimated glomerular filtration rate
ERK	extracellular signal-regulated kinase
ESRD	end-stage renal disease
FcRn	neonatal fragment crystallizable (Fc) receptor
FSGS	focal segmental glomerulosclerosis
GBM	glomerular basement membrane
Gd-IgA1	aberrantly glycosylated IgA1
GFR	glomerular filtration rate
GP	glomerulopathy
GPx	glutathione peroxidase
GR	glutathione reductase
GSH	glutathione
HMOX1	heme oxygenase-1
HTN	hypertension
hs-CRP	high-sensitivity C-reactive protein
IgAN	IgA nephropathy
IL-6	interleukin 6
IRF1	interferon regulatory factor 1
JNK	jun-N-terminal kinase
Keap1	Kelch-like ECH-associated protein 1
LDL	low-density lipoproteins
LOX	lipoxygenase
MAPK	mitogen-activated protein kinases
MCD	minimal change disease
MDA	malondialdehyde
MN	membranous nephropathy/glomerulopathy
MPO	myeloperoxidase
NADH	nicotinamide adenine dinucleotide hydrogen
NADPH	nicotinamide adenine dinucleotide phosphate
NF-κB	nuclear factor kB
NO	nitric oxide
NOX	NADH/NADPH oxidase
Nrf2	nuclear factor (erythroid-derived 2)-like 2
OxSt	oxidative stress
PAN	puromycin aminonucleoside nephrosis
PARP	poly(ADP-ribose)polymerase
PKC	protein kinase C
RAAS	renin-angiotensin-aldosterone system
RAGE	receptor for advanced glycation end products
Smad3	mothers against decapentaplegic homolog 3
SOD	superoxide dismutase
ROS	reactive oxygen species
RNS	reactive nitrogen species
tBid	truncated BH3 interacting domain death agonist
TGF-β	transforming growth factor-beta
TNF-α	tumor necrosis factor-alpha
TRPV1	transient receptor potential vanilloid channel 1
UrOb	ureteral obstruction
XO	xanthine oxidase
